# New Books

**Published:** 2008-05

**American Hegemony and the Postwar Reconstruction of Science in Europe**

John Krige

Cambridge, MA:MIT Press, 2008. 392 pp. ISBN: 978-0-262-61225-8, $23

**Blue Covenant**

Maude Barlow

New York:New Press, 2007. 208 pp. ISBN: 978-1-59558-186-0, $24.95

**CO2** **Rising: The World’s Greatest Environmental Challenge**

Tyler Volk

Cambridge, MA:MIT Press, 2008. 264 pp. ISBN: 78-0-262-22083-5, $22.95

**Energy for Sustainability: Technology, Planning, Policy**

John Randolph, Gilbert Masters

Washington, DC:Island Press, 2008. 816 pp. ISBN: 1-59726-103-3, $85

**Energy in Nature and Society: General Energetics of Complex Systems**

Vaclav Smil

Cambridge, MA:MIT Press, 2008. 512 pp. ISBN: 978-0-262-69356-1, $32

**Environmental Policy Analysis and Practice**

Michael R. Greenberg

Piscataway, NJ:Rutgers University Press, 2008. 304 pp. ISBN: 978-0-8135-4276-8, $32.95

**Essential Concepts in Toxicogenomics**

Donna L. Mendrick, William B. Mattes, eds.

Totowa, NJ:Humana Press, 2008. 325 pp. ISBN: 978-1-58829-638-2, $99.50

**Fuel**

John Knechtel, ed.

Cambridge, MA:MIT Press, 2008. 320 pp. ISBN: 978-0-262-11325-0, $15.95

**Genomics Protocols, 2nd ed.**

Mike Starkey, Ramnath Elaswarapu, eds.

Totowa, NJ:Humana Press, 2008. 532 pp. ISBN: 978-1-58829-871-3, $99.50

**Global Catastrophes and Trends: The Next Fifty Years**

Vaclav Smil

Cambridge, MA:MIT Press, 2008. 320 pp. ISBN: 978-0-262-19586-7, $29.95

**Lake Effect: Two Sisters and a Town’s Toxic Legacy**

Nancy A. Nichols

Washington, DC:Island Press, 2008.144 pp. ISBN: 1-59726-084-3, $25

**Nuclear Power Is Not the Answer**

Helen Caldicott

New York:New Press, 2007. 240 pp. ISBN: 978-1-59558-213-3, $15.95

**Perspectives on Sustainable Resources in America**

Roger A. Sedjo, ed.

Washington, DC:RFF Press, 2008. 300 pp. ISBN: 978-1-933115-63-4, $80

**Poisoned for Pennies: The Economics of Toxics and Precaution**

Frank Ackerman

Washington, DC:Island Press, 2008. 328 pp. ISBN: 1-59726-400-8, $50

**Scientific Collaboration on the Internet**

Gary M. Olson, Ann Zimmerman, Nathan Bos, eds.

Cambridge, MA:MIT Press, 2008. 432 pp. ISBN: 978-0-262-15120-7, $45

**Scientists Debate Gaia: The Next Century**

Stephen H. Schneider, James R. Miller, Eleen Crist, Penelope J. Boston, eds.

Cambridge, MA:MIT Press, 2008. 400 pp. ISBN: 978-0-262-69369-1, $30

**Taming the Anarchy: Groundwater Governance in South Asia**

Tushaar Shah

Washington, DC:RFF Press, 2008. 300 pp. ISBN: 978-1-933115-60-3, $49.95

**The Shadows of Consumption: Consequences for the Global Environment**

Peter Dauvergne

Cambridge, MA:MIT Press, 2008. 328 pp. ISBN: 978-0-262-04246-8, $24.95

**Water, Place, and Equity**

John M. Whiteley, Helen Ingram, Richard Warren Perry, eds.

Cambridge, MA:MIT Press, 2008. 312 pp. ISBN: 978-0-262-23271-5, $63

